# Levels of autonomy in synthetic biology engineering

**DOI:** 10.15252/msb.202010019

**Published:** 2020-12-17

**Authors:** Jacob Beal, Miles Rogers

**Affiliations:** ^1^ Raytheon BBN Technologies Cambridge MA USA

## Abstract

Engineering biological organisms is a complex, challenging, and often slow process. Other engineering domains have addressed such challenges with a combination of standardization and automation, enabling a divide‐and‐conquer approach to complexity and greatly increasing productivity. For example, standardization and automation allow rapid and predictable translation of prototypes into fielded applications (e.g., “design for manufacturability”), simplify sharing and reuse of work between groups, and enable reliable outsourcing and integration of specialized subsystems. Although this approach has also been part of the vision of synthetic biology, almost since its very inception (Knight & Sussman, 1998), this vision still remains largely unrealized (Carbonell *et al*, 2019). Despite significant progress over the last two decades, which have for example allowed obtaining and editing DNA sequences in easier and cheaper ways, the full process of organism engineering is still typically rather slow, manual, and artisanal.

## Graphical abstract

Engineering biological organisms is a complex process and challenging that could benefit from a combination of standardization and automation. This Commentary discusses the advantages and challenges of achieving high levels of autonomy in synthetic biology.

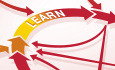

Perhaps it is time to take a more systematic approach to automation in organism engineering, to better understand the barriers to productivity gains. In electrical, mechanical, and chemical engineering, where automation and high productivity have become the norm, the success has come from breaking down complex processes into simple, well‐understood steps in a precisely managed environment. However, when engineering living organisms, we are dealing with complex and imperfectly understood systems that cannot be so easily controlled. It may therefore be more helpful to think beyond automation to *autonomy*. While specific definitions of autonomy vary (e.g., Beer *et al*, 2014; Kaber, 2018), the general theme is that automation is any machine taking over actions from a human, while autonomy is automation operating with resilience and independence in a complex open environment.

## Levels of autonomy

We would like to begin by proposing a working definition of autonomy for synthetic biology engineering, to use both for evaluating the degree of autonomy offered by current systems and for considering options for future development. While there is a wide variety of definitions and frameworks regarding autonomy, we propose that a particularly well suited framework to adapt is the Levels of Driving Automation (LoDA) framework, which was developed by SAE International and is now widely used throughout the automotive engineering community. This framework consists of six levels (0 through 5) of incrementally increasing autonomy. The lower three levels in the LoDA framework, that is, no automation, driver assistance, and partial automation, can be met by isolated subsystems supervised by the human driver. The higher levels, however—conditional automation, high automation, and full automation—require that the system be fully integrated and capable of closed loop operation.

By analogy, we may consider a synthetic biology investigator as the “driver” of a laboratory, and the collection of assistive equipment therein the vehicle that the investigator navigates toward an intended organism engineering goal. A laboratory, of course, does not exist in a vacuum, but makes use of externally supplied reagents, instruments, protocols, etc. Such dependencies do not disrupt the notion of autonomy, but merely imply additional standards and compatibility requirements, just as with a vehicle's analogous use of gasoline, automotive parts, satellite navigation, and so on.

**Figure 1:**
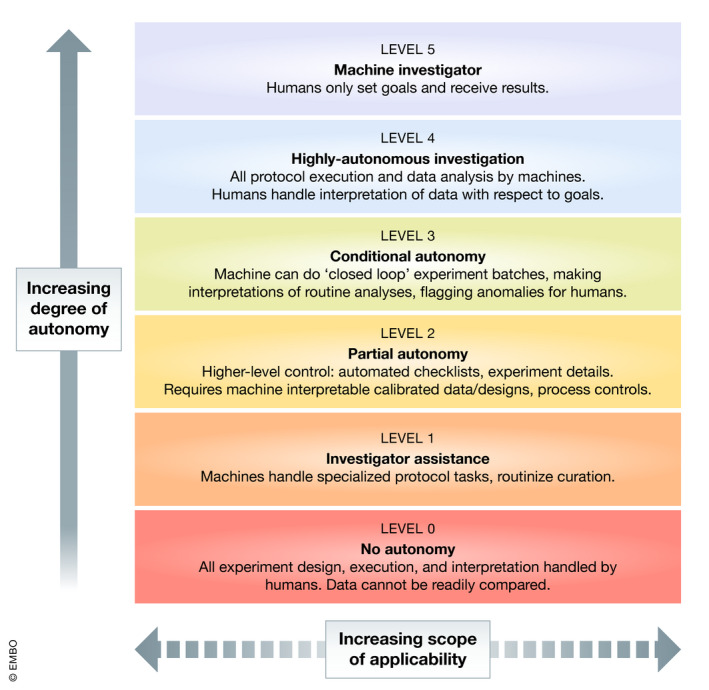
**Levels of autonomy in synthetic biology workflows, incrementally rising from no autonomy to fully autonomous machine investigation**.

Figure 1 illustrates our proposed levels of autonomy for synthetic biology, adapting the concepts from the SAE LoDA to this domain. The primary axis is the six vertical levels of increasing degree of autonomy:

- Level 0, No Autonomy: No autonomy is the current condition of most work in most laboratories, with essentially all work carried out by humans.
- Level 1, Investigator Assistance: The first level of autonomy introduces narrowly scoped systems assisting with specific labor‐intensive tasks, such as “high‐throughput” assay instruments, pipetting robots, or specialized software packages. However, humans are still intimately entangled with their operation, which typically requires careful set‐up for each task to be executed.
- Level 2, Partial Autonomy: Partially autonomous systems provide proactive assistance to the investigator. For example, a system may validate its operation against a checklist of potential problems or fill in details of a more abstract experiment plan. Since the system needs to reason about its operations, this level also requires increased use of standards, calibration, process controls, and extraction of “intuitive” knowledge about the task into a machine‐interpretable form.
- Level 3, Conditional Autonomy: The third level of autonomy marks a major transition, in which the machine is able to close the design‐build‐test‐loop, running multiple cycles without human intervention, beginning to interpret routine analyses, and involving humans only in case of anomalies and at the completion of a batch. This means that all individual workflow components must be at least Level 2, and they also must be integrated and able to adapt to results from other parts of the workflow. For example, if an automatically designed construct fails in the build or test stages, the next iteration of design should adapt at least enough to not propose the same construct again.
- Level 4, Highly Autonomous Investigation: At the fourth level, the system is essentially a laboratory assistant, taking over all protocol execution and routine aspects of data analysis, while the human is still required for interpreting data with respect to goals and adjusting plans accordingly.
- Level 5, Machine Investigator: At this highest level, the human moves from investigator to manager, essentially removing themselves from laboratory operations except for setting goals and receiving results.

Orthogonally, we may also consider the scope of a system’s applicability. At the lower levels, scope may refer to how much of a workflow is covered by a system. Scope may also refer to the system's versatility in applicability: a narrowly scoped system might only apply a certain test protocol, while a more broadly scoped system might apply to a wide range of build or test protocols.

This does not necessarily mean that we expect to achieve all of these levels. For example, Level 5 autonomy might well likely require rather sophisticated Artificial Intelligence. Nevertheless, having this framework in hand will allow a more quantitative assessment of the current state‐of‐the‐art and will indicate key barriers to improved productivity.

## State‐of‐the‐Art

A number of projects have demonstrated that high levels of autonomy are indeed possible in synthetic biology. For example, Level 3 autonomy has been demonstrated with organic synthesis via an integrated fluidic system and machine learning classifier (Granda *et al*, 2018) and in other chemical investigations via a mobile robotic system and Bayesian sample design (Burger *et al*, 2020). The “Adam” and “Eve” robotic science systems (King *et al*, 2009) arguably attain Level 4 autonomy, via systems biology knowledge representations that allow both experiment configuration from mechanistic hypotheses and hypothesis adjustment from results.

However, just as with early demonstrations of autonomous vehicle navigation, there is a sizable gap between demonstrating that high‐level autonomy is possible and actually increasing the level of autonomy that is broadly deployed. These demonstrations, while impressive, are still fragile, narrow in scope, and require considerable prior investment in configuration and curation to set up an experimental program. Returning once again to the vehicle analogy, the prior systems are all still driving on a closed test course and not the open and unpredictable urban environment of most synthetic biology research.

**Figure 2:**
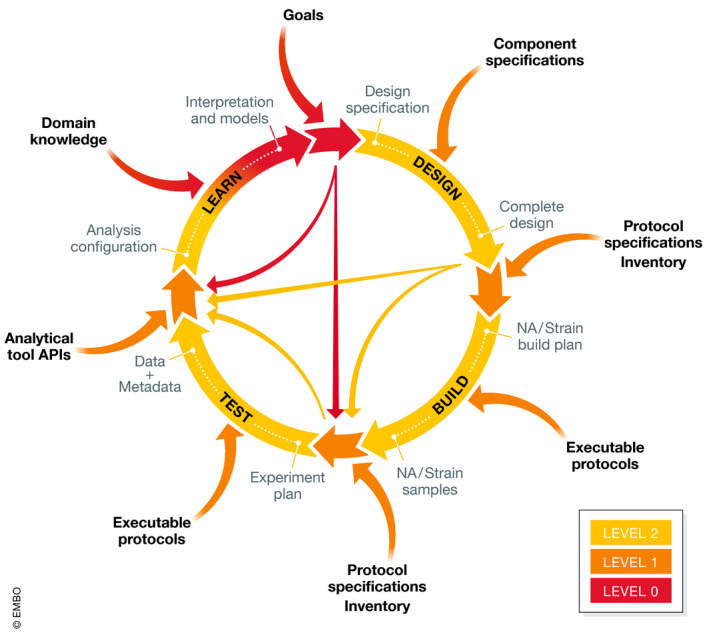
State of the art in autonomy for design‐build‐test‐learn cycle. State‐of‐the‐art in synthetic biology autonomy, showing both the core design‐build‐test‐learn cycle and also the configuration required to connect between stages and the curation required enable autonomy for each stage and its configuration. Color indicates maximum autonomy level available via publicly available reusable components, per the levels shown in Figure 1. At best, current systems are attaining partial autonomy (Level 2) in isolated portions of the cycle, with major gaps regarding stage‐to‐stage connections and curation.

At present, any automation is generally provided at the level of components and partial systems. The commonly discussed notion of a design‐build‐test‐learn engineering cycle can be useful in understanding the challenges in moving from the current state‐of‐the‐art to a more generally available high‐level autonomy. Figure 2 illustrates this cycle, coloring components by the highest level of broadly available automation. It also includes two other commonly elided aspects: configuration and curation. Configuration connects the output from one engineering phase to the inputs of another engineering phase, such as setting up a test phase experiment using genetic constructs from a build phase plus information about their design intention and hypotheses from the preceding design and learn phases. Curation provides the machine‐interpretable information required for both execution and configuration, such as protocols to be used in a test and the inventory of available equipment, constructs, strains, and reagents.

Significant automation capabilities have been developed for each of the four primary phases: design, build, test, and learn. At least in some domains, there are systems providing Level 2 partial autonomy, such that a well‐configured system can perform significant reasoning about tasks it executes, validate its operation, and provide meaningful debugging assistance to a human operator.

**Figure 3:**
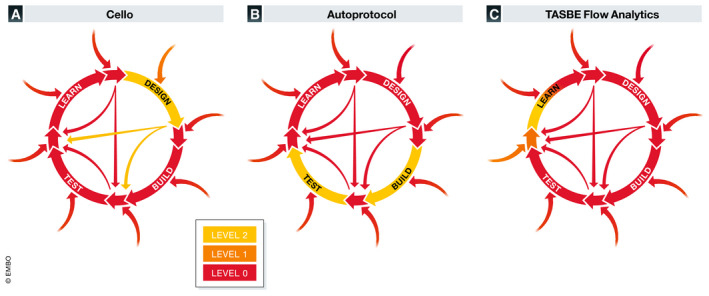
Examples of level of autonomy analysis for synthetic biology tools. Example level of autonomy analysis for state‐of‐the‐art partially autonomous (Level 2) systems in synthetic biology, indicating level by color as in Fig 1: (A) Cello (Nielsen *et al*, 2016) genetic circuit design software, (B) Autoprotocol (Miles & Lee, 2018) for build and test protocol automation, and (C) TASBE Flow Analytics (Beal *et al*, 2019) for learning from analysis of flow cytometry data. Note that collectively such systems cover nearly all of the design‐build‐test‐loop, but are effectively isolated in current practice by gaps in curation and configuration.

For example, Cello (Nielsen *et al*, 2016) (Fig 3A) automates the design of a class of genetic information processing circuits, given a specification of a desired logic function and library of device models, using those models to guide design and predict expected behavior. Notably, while Cello is not the first tool that in principle allows such a design process—others include BioCompiler, GEC, and GenoCAD—the success of Cello circuit design is largely due to its creators' curation of library of high‐quality device characterization data, which is still a largely manual process with at best Level 1 automation. Similarly, Autoprotocol (Miles & Lee, 2018) (Fig 3B) provides automation for build and test protocol execution. However, authoring new protocol scripts is a complex process often requiring significant expertise and multiple iterations. The same authoring challenge exists with all other current protocol automation platforms, such as Aquarium, Antha, and the OpenTrons API. In the learn phase, we find analysis packages like TASBE Flow Analytics (Beal *et al*, 2019) (Fig 3C), which carries out automated processing and quality control assessment of flow cytometry data, using heuristics and a checklist of common issues to effectively implement partial autonomy in analysis. Similar automation exists for other assays, such as the Galaxy workflow environment for bioinformatics and omics, or microscopy packages such as SuperSegger and FogBank. Nevertheless, organizing data for analysis is largely manual, and interpretation of results with respect to background knowledge and experimental goals is still left entirely to human experts.

In sum, while automation is available for any given phase, the current state‐of‐the‐art consistently falls short when it comes to the configuration of phase‐to‐phase interconnections and curation of information to satisfy preconditions for execution. Some representational standards have already been developed to address configuration and curation challenges, for example, SBML for composable models of learned information, SBOL for linking between design information and the rest of the engineering cycle, and workflow integrations have been demonstrated making use of these standards.

Nevertheless, three major gaps still remain unaddressed. First, there is not yet any standard representation for protocols and protocol interfaces, complementary to SBOL and SBML. Such a representation is needed for configuration of build and test automation within larger workflows. Second, curation of information into standard representations—a precondition for automation—is still a slow and highly manual process, requiring rare joint expertise in both knowledge representations and the particular application domain targets of curation. Level 2 (partial autonomy) tools are needed in order to act as assistive partners to domain experts, thereby lowering the barriers to curation and decreasing the need for knowledge representation expertise. Finally, there is not yet a critical mass of automation‐enabled tools to form an effective marketplace for automated workflows. While such a marketplace effectively exists within the learn phase for bioinformatics tooling, a sufficiency of Level 1 and Level 2 automation tools in other domains need to be adapted to use standard representations in order to facilitate workflow integration by non‐specialists across larger portions of the engineering cycle.

In sum, at present, autonomy in synthetic biology has been demonstrated as high as Level 4 (highly autonomous investigation). While higher levels of autonomy should benefit investigators by allowing faster and more effective engineering, nearly all investigations are still minimally automated, at best having only fragments of a workflow even as high as Level 2 (partial autonomy).

## From autonomy to society

The gaps noted above, protocol representation, lightweight curation, and automation Markets, all point to the critical role that open standards must play in enabling autonomy in synthetic biology. For any business involving technological innovation, Joy's Law is the principle that: “No matter who you are, most of the smartest people work for someone else.” This reflects the fact that the expertise needed for complex problems is diverse, highly specialized, and difficult to transfer. Research and development in synthetic biology is a particular extreme in this respect, given its wildly diverse and interdisciplinary nature. Thus, unlike autonomous driving, high‐level autonomy in synthetic biology is a problem that needs to be solved not once but countless times, as every laboratory has its own particular set of needs, goals, protocols, and available equipment.

Closed or bespoke systems, like those that have achieved high levels of autonomy in the past, simply cannot bring to bear the level of marketplace resources that can be marshaled with the aid of open standards. Once a critical mass is reached, network effects will incentivize widespread adoption of standards, as investigators make use of that marketplace to obtain a competitive edge and as tool providers aim for sales within it. At that point, we should expect an exponential take‐off in the impact of synthetic biology and a wholesale global transformation, much as with the manufacturing revolution set off by electrical standards at the end of the 19^th^ century and the informational revolution set off by computer network standards at the end of the 20^th^ century.

Until that point, however, the field is in a tentative state, vulnerable to being stifled by monopolistic capture (intentional or emergent), by rent‐taking outside forces, or by intellectual property gridlock. The synthetic biology community thus needs to make active choices to promote markets based on open biological standards. Individual practitioners should look for ways to gain advantage through becoming early adopters. Companies should join open standards consortia or create them in those areas where none exist at present. Investors should look for ways to leverage standards in market plays. Finally, professional societies should advocate for funding supporting open standards, and government agencies and program managers should explicitly support standards development and adoption within their portfolios.

In the coming decades, those societies that invest in open standards for synthetic biology will experience vast gains in biological productivity and the corresponding economic and societal benefits. It behooves all of us to look for ways that we can contribute to bringing that future into being.

## Conflict of interest

The authors declare that they have no conflict of interest.

**Box 1: Further reading**

1. SAE levels of driving automation

SAE International (2016) Taxonomy and definitions for terms related to driving automation systems for on‐road motor vehicles. Technical Report J 3016 201609, SAE International.

- Automation methods

Afgan E, Baker D, Batut B, Van Den Beek M, Bouvier D, Čech M, Chilton J, Clements D, Coraor N, Grüning BA *et al* (2018) The galaxy platform for accessible, reproducible and collaborative biomedical analyses: 2018 update. *Nucleic Acids Res* 46(W1): W537–W544

Beal J, Lu T, Weiss R (2011) Automatic compilation from high‐level biologically‐oriented programming language to genetic regulatory networks. *PLoS ONE* 6: e22490

Chalfoun J, Majurski M, Dima A, Stuelten C, Peskin A, Brady M (2014) Fogbank: a single cell segmentation across multiple cell lines and image modalities. *BMC Bioinformatics* 15: 431

Czar MJ, Cai Y, Peccoud J (2009) Writing DNA with GenoCAD. *Nucleic Acids Res* 37(Web Server issue): W40–W47

Pedersen M, Phillips A (2009) Towards programming languages for genetic engineering of living cells. *J Roy Soc Interf Roy Soc* 6: S437–S450

Stylianidou S, Brennan C, Nissen SB, Kuwada NJ, Wiggins PA (2016) Supersegger: robust image segmentation, analysis and lineage tracking of bacterial cells. *Mol Microbiol* 102: 690–700

- Standards and Integration

Hucka M, Finney A, Sauro HM, Bolouri H, Doyle JC, Kitano H, Arkin AP, Bornstein BJ, Bray D, Cornish‐Bowden A *et al* (2003) The Systems Biology Markup Language (SBML): a medium for representation and exchange of biochemical network models. *Bioinformatics* 19: 524–531

Myers CJ, Beal J, Gorochowski TE, Kuwahara H, Madsen C, McLaughlin JA, Misirli G, Nguyen T, Oberortner E, Samineni M *et al* (2017) A standard‐enabled workflow for synthetic biology. *Biochem Soc Trans* 45: 793–803

Roehner N, Beal J, Clancy K, Bartley B, Misirli G, Grnberg R, Oberortner E, Pocock M, Bissell M, Madsen C *et al* (2016) Sharing structure and function in biological design with SBOL 2.0. *ACS Synth Biol* 5: 498–506
